# Electrical, electrochemical and structural studies of a chlorine-derived ionic liquid-based polymer gel electrolyte

**DOI:** 10.3762/bjnano.12.92

**Published:** 2021-11-18

**Authors:** Ashish Gupta, Amrita Jain, Manju Kumari, Santosh K Tripathi

**Affiliations:** 1 Department of Physics, Government Tulsi Degree College, Anuppur, Madhya Pradesh, 484224, India; 2 Institute of Fundamental Technological Research, Polish Academy of Sciences, Adolfa Pawińskiego 5b, 02-106, Warsaw, Poland; 3 Viva Institute of Technology, Shirgaon, Virar East, Maharastra, 401305, India; 4 Department of Physics, School of Physical Sciences, Mahatma Gandhi Central University, Bihar-845401, India

**Keywords:** ionic liquid, polymer gel electrolytes, solution casting technique, transference number

## Abstract

In the present article, an ionic liquid-based polymer gel electrolyte was synthesized by using poly(vinylidene fluoride-*co*-hexafluoropropylene) (PVdF-HFP) as a host polymer. The electrolyte films were synthesized by using the solution casting technique. The as-prepared films were free-standing and transparent with good dimensional stability. Optimized electrolyte films exhibit a maximum room-temperature ionic conductivity of σ = 8.9 × 10^−3^ S·cm^−1^. The temperature dependence of the prepared polymer gel electrolytes follows the thermally activated behavior of the Vogel–Tammann–Fulcher equation. The total ionic transference number was ≈0.91 with a wider electrochemical potential window of 4.0 V for the prepared electrolyte film which contains 30 wt % of the ionic liquid. The optimized films have good potential to be used as electrolyte materials for energy storage applications.

## Introduction

For the past two decades, researchers have been developing polymer electrolytes (solid/gel) as an alternative to commercial liquid-based electrolytes which are suitable for electrochemical devices, such as Li-ion batteries, solar cells, fuel cells, and supercapacitors [1–5]. The main aim is to increase the amorphous content in the polymer which assists in the rapid ion motion while keeping its mechanical stability. The second aim is to increase the ionic conductivity of the electrolytes, which is generally insufficient for practical applications in electrochemical energy storage devices. Hence, different kinds of techniques, such as the addition of ionic liquids (ILs) with low viscosity and high dielectric constant values or some suitable fillers have been used by the research community to increase the ionic conductivity of polymer electrolytes [6–7].

As mentioned above, one way to increase the ionic conductivity is to introduce ILs into polymer gel electrolytes. Currently, ILs have received much attention due to their unique properties, such as a wide electrochemical window, high ionic conductivity, non-volatility, non-flammability, small vapor pressure, broad liquid range, and outstanding thermal stability in the range of 200–300 °C [8–11]. Basically, ILs are room-temperature molten salts made up of bulky asymmetric organic cations and organic/inorganic anions. They act as plasticizers which help increase the amorphous nature of the polymer gel electrolytes, thereby increasing the ionic conduction [12–13]. There are various studies on halide-based ILs used in polymer electrolytes which are reported in the literature [14–16]. However, there are limited reports available in the literature on polymer gel electrolytes incorporating chloride-based ILs. Attri et al. [17] synthesized 1-butyl-2,3-dimethylimidazolium chloride ([BDiMIM][Cl]), tested its application in polymer electrolytes, and obtained a maximum room-temperature conductivity of 2.7 × 10^−6^ S·cm^−1^.

Among various polymer matrix systems, such as polyethylene oxide (PEO) [18–19], polyvinyl alcohol (PVA) [20–21], and polyvinylpyrrolidone (PVP) [22–23], fluoropolymers such as poly(vinylidene fluoride-*co*-hexafluoropropylene) (PVdF-HFP) have received much attention from the research community as potential polymer hosts for the synthesis of polymer electrolytes [10–11,24–25]. The dielectric constant value of PVdF-HFP is ≈8.4 and it comprises a predominantly crystalline PVdF phase and an amorphous HFP phase, which provides necessary mechanical strength and good ion transport matrix. Magnesium-based electrochemical devices are emerging as an alternative to lithium-based devices [26–30]. Magnesium can be an alternative due to its availability in the Earth’s crust, low atomic weight, low price, high electrochemical reduction potential of −2.3 V versus the standard hydrogen electrode. Moreover, most of the Mg compounds are usually nontoxic and also Mg is chemically stable as compared to lithium and sodium [31].

In the present study, the ionic liquid [BDiMIM][Cl], the host polymer PVdF-HFP, and the salt magnesium perchlorate (Mg(ClO_4_)_2_) were used to prepare polymer electrolyte films. The synthesized films were characterized by different microscopic and electrochemical techniques.

## Results and Discussion

### Structural characterization

**Figure 1:**
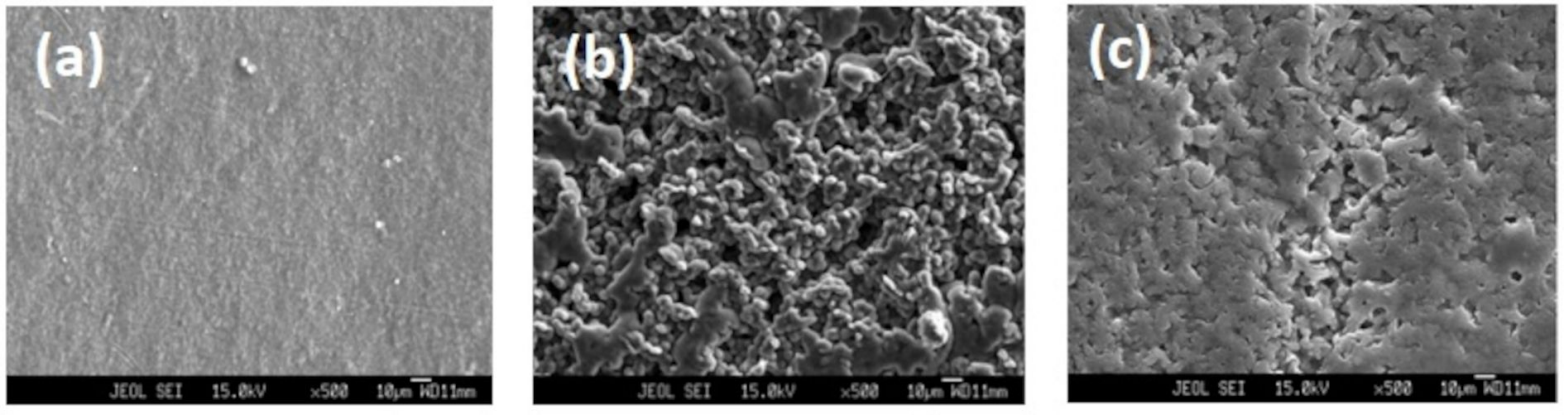
SEM images of (a) PVdF-HFP, (b) {(PVdF-HFP)-[BDiMIM][Cl]} (4:6), and (c) ({(PVdF-HFP)-[BDiMIM][Cl]} (4:6)) (20 wt %) + [PC-Mg(ClO_4_)_2_ (0.3 M)] (80 wt %) films.

The surface morphology of a pure PVdF-HFP film, {(PVdF-HFP)-[BDiMIM][Cl]} (4:6), and ({(PVdF-HFP)-[BDiMIM][Cl]} (4:6)) (20 wt %) + [PC-Mg(ClO_4_)_2_ (0.3 M)] (80 wt %), where PC is the plasticizer propylene carbonate, was characterized using scanning electron microscopy (SEM) and is depicted in Figure 1a–c. Figure 1a shows the surface morphology of a pure PVdF-HFP film, indicating an uneven and rough surface of the crystalline polymer.

Upon the addition of the ionic liquid [BDiMIM][Cl] on PVdF-HFP, the sizes of the grains start decreasing and the membrane becomes flexible and appears as a swollen structure (Figure 1b). Furthermore, when the mixture of PC-Mg(ClO_4_)_2_ is added to the film, it becomes more amorphous with no crystalline grains and no dissolved salts (Figure 1c). The smoothness of the film suggests the amorphous nature of electrolyte films. Similar morphology features were obtained in our previous work [10]. The uniformly distributed pores present in the films lead to the retention of liquid electrolytes, thereby increasing the overall ionic conductivity of the electrolyte films.

**Figure 2:**
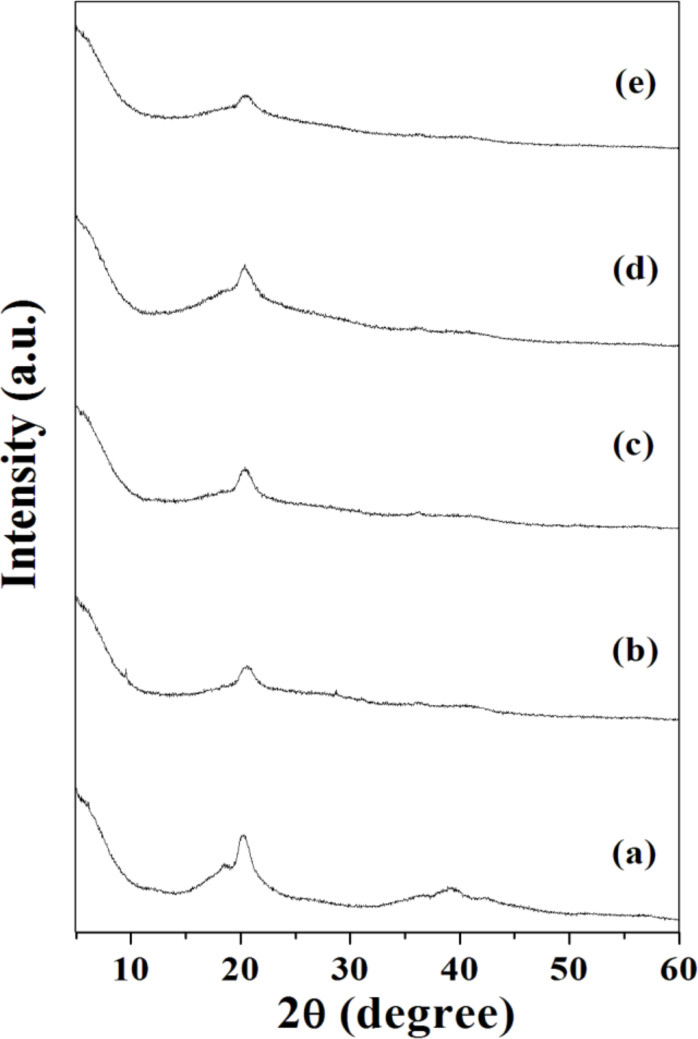
XRD spectra of (a) a pure PVdF-HFP film, (b) {(PVdF-HFP)-[BDiMIM][Cl]} (4:6), (c) {(PVdF-HFP)-[BDiMIM][Cl]} (3:7), (d) ({(PVdF-HFP)-[BDiMIM][Cl]} (4:6)) (20 wt %) + [PC-Mg(ClO_4_)_2_ (0.3 M)] (80 wt %), and (e) ({(PVdF-HFP)-[BDiMIM][Cl]} (4:6)) (30 wt %) + [PC-Mg(ClO_4_)_2_ (0.3 M)] (70 wt %) polymer gel electrolytes films.

X-ray diffraction (XRD) patterns were also recorded to check the crystallinity of the synthesized material. The variations in the crystallinity of the polymer electrolytes are directly related to the intensity and full width at half maximum (FWHM) of the peak. Usually, the crystallinity and FWHM are inversely proportional to each other, the larger the FWHM, the smaller the crystal size and vice versa [32]. XRD patterns of the electrolyte membranes are depicted in Figure 2a–e.

The XRD pattern of pure PVdF-HFP can be marked according to the α, β, and γ PVdF crystalline phases. From Figure 2a, it can be seen that pure PVdF-HFP possesses well-defined peaks at 18.4, 20.4, 25.6, and 38°. The peak at 18.4° [20] corresponds to the PVdF α-phase and the broad peaks at 20.4 and 25.6° show the superposition of the β- and γ-phases, respectively [33].

Furthermore, it can be observed from the plot that after mixing the polymer with the ionic liquid [BDiMIM][Cl] and by dissolving the liquid electrolyte PC-Mg(ClO_4_)_2_ solution in it, some peaks of PVdF-HFP at 2θ = 38° disappear [10]. This indicates that the ionic liquid as well as the liquid electrolyte solution form a complete complexation with the polymer PVdF-HFP at a molecular level in polymer gel electrolytes. Additionally, the broadening of the XRD peak at 2θ = 20.4° suggests that by mixing an ionic liquid and a liquid electrolyte solution with a pure polymer the amorphization increases, leading to the enhancement of the ionic conductivity of the polymeric system. Moreover, the diffraction peaks of polymer gel electrolytes have relatively lower intensities as compared to a pure polymer, which indicates that the ionic liquid and the liquid electrolytes have good compatibility with each other.

The different parameters such as *d*-spacing, crystallite size/Scherrer length (*l*), relative peak intensity of the polymers, pure salt content, polymer/ionic liquid blend, and polymer gel electrolytes were calculated from the XRD plot by using the Debye–Scherrer equation [34], which is given by Equation 1:


[1] $$d=\frac{K\lambda }{\beta \cos\theta },$$

where *d* is the size of the crystal, *K* is the constant, λ is the X-ray wavelength at 1.54 Å for Cu Kα radiation, β is the full width at half maximum of the peak (in radians), and θ is the Bragg angle (in degrees). The calculated results are tabulated in Table 1.

**Table 1:** Values of the parameters obtained from X-ray diffraction of ionic liquid-based polymer gel electrolytes.

Sample	2θ (deg)	θ	*d*(Å)	*l*(Å)	Intensity

host polymer	20.4	10.2	4.52	0.22	100
[polymer:IL] (4:6)	20.6	10.3	4.3	0.11	100
[polymer:IL] (3:7)	20.4	10.2	4.5	0.14	100
[polymer:IL (4:6)] (20 wt %)-[liquid electrolyte (0.3 M) (80 wt %)]	20.4	10.2	4.4	0.12	100
optimized composition	20.6	10.3	4.1	0.08	100

**Figure 3:**
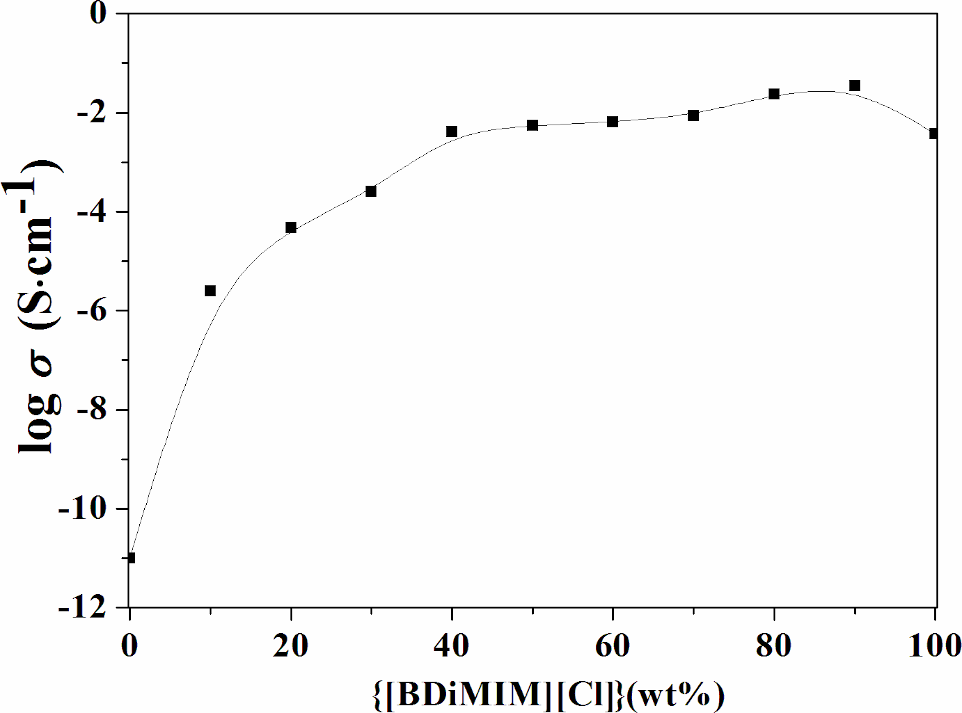
Room-temperature electrical conductivity of the polymer/ionic liquid blend as a function of the ionic liquid concentration.

As it can be seen from the results obtained from SEM and XRD, the addition of a liquid electrolyte in the host polymer significantly decreases the crystallinity of the latter, which is expected to result in an increase of the ionic conductivity of electrolyte films. To confirm this, the variation of the room-temperature ionic conductivity (σ) of the polymer/ionic liquid (PVdF-HFP)-[BDiMIM][Cl] blend as a function of the ionic liquid [BDiMIM][Cl] concentration is shown in Figure 3.

Initially, the conductivity of the host polymer PVdF-HFP was measured to be in the order of ≈10^−10^ S·cm^−1^ at room temperature. Upon the addition of the ionic liquid [BDiMIM][Cl] in the host polymer PVdF-HFP, the conductivity of the system significantly increased and reached the maximum value of 6.5 × 10^−3^ S·cm^−1^ at 60 wt % of the ionic liquid content. Beyond 60 wt %, it was found that the conductivity remained constant and the film was dimensionally unstable. The increase in conductivity might be due to the combined effect of plasticization (responsible for an enhanced flexibility of the polymer backbone) and generation of free mobile charged species, namely [BDiMIM]^+^ and [Cl]^−^ in a PVdF-HFP ionic liquid blend system. Moreover, this increase might also be due to a higher probability of H-bonding between protic IL and the polymer affecting its conductivity in a positive way. Usually, this change in conductivity pattern is due to the involvement of π-electrons responsible for the generation of charge carriers and their switching, which results in a reduction of the electron density of the overall system and an increase in the activation energy [10]. Simultaneously, there is also a decrease in the activation energy produced by an extended intermolecular overlap, which might also be due to H-bonding. In other words, an increase in the number of electrons associated in the H-bonding might be the reason of an increased ionic conductivity of ionic liquid-based systems [17].

**Figure 4:**
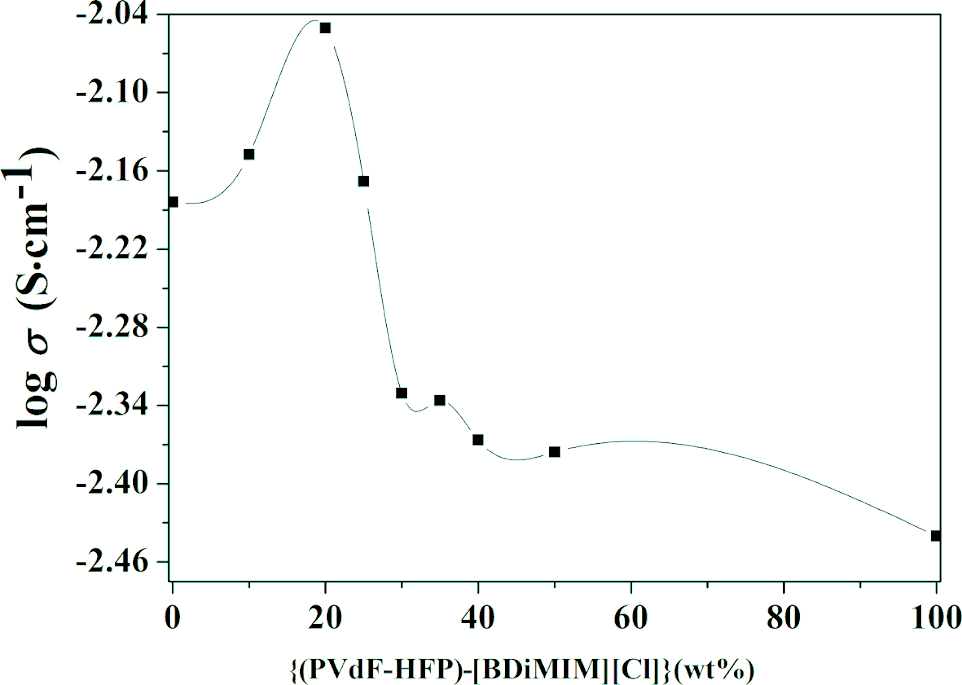
Variation of the electrical conductivity of a polymer gel electrolyte as a function of the polymer/ionic liquid blend concentration.

Finally, {(PVdF-HFP) (40 wt %)-[BDiMIM][Cl] (60 wt %)} was chosen as the optimized blend. After the optimization of polymer and ionic liquid blends, a liquid electrolyte was introduced in the system. Accordingly, different weight percentages of the polymer/ionic liquid blend, {(PVdF-HFP)-[BDiMIM][Cl]} (4:6), in the solution of an optimized liquid electrolyte PC-Mg(ClO_4_)_2_ (0.3 M) system were tested and the optimization curve is shown in Figure 4.

As it can be seen from Figure 4, the maximum conductivity of the liquid electrolyte PC-Mg(ClO_4_)_2_ at room temperature is found to be ≈3.36 × 10^−3^ S·cm^−1^. Upon the addition of different weight percentages of a polymer/ionic liquid blend {(PVdF-HFP)-[BDiMIM][Cl]} (4:6) in an optimized composition of liquid electrolytes PC-Mg(ClO_4_)_2_ (0.3 M), the ionic conductivity of the polymer gel electrolyte initially increases and reaches its maximum value at 20 wt % (σ = 8.9 × 10^−3^ S·cm^−1^) and thereafter it starts to decrease. It can be clearly seen from the conductivity plot that relatively higher conductivity values of polymer gel electrolytes were obtained as compared to the liquid electrolyte, which can be explained on the basis of a breathing chain model proposed by Chandra et al. [35]. The decrease in conductivity values after 20 wt % of the (PVdF-HFP)-IL blend is due to the increased viscosity of the electrolyte system, which lowers the mobility of mobile species and hence the conductivity decreases thereafter. Finally, ionic liquid-based polymer gel electrolytes with an optimized composition of ({(PVdF-HFP)-[BDiMIM][Cl]} (4:6)) (20 wt %) + [PC-Mg(ClO_4_)_2_ (0.3 M)] (80 wt %) were chosen for detailed studies. The maximum room-temperature ionic conductivity of the optimized system was found to be in the order of ≈8.9 × 10^−3^ S·cm^−1^.

**Figure 5:**
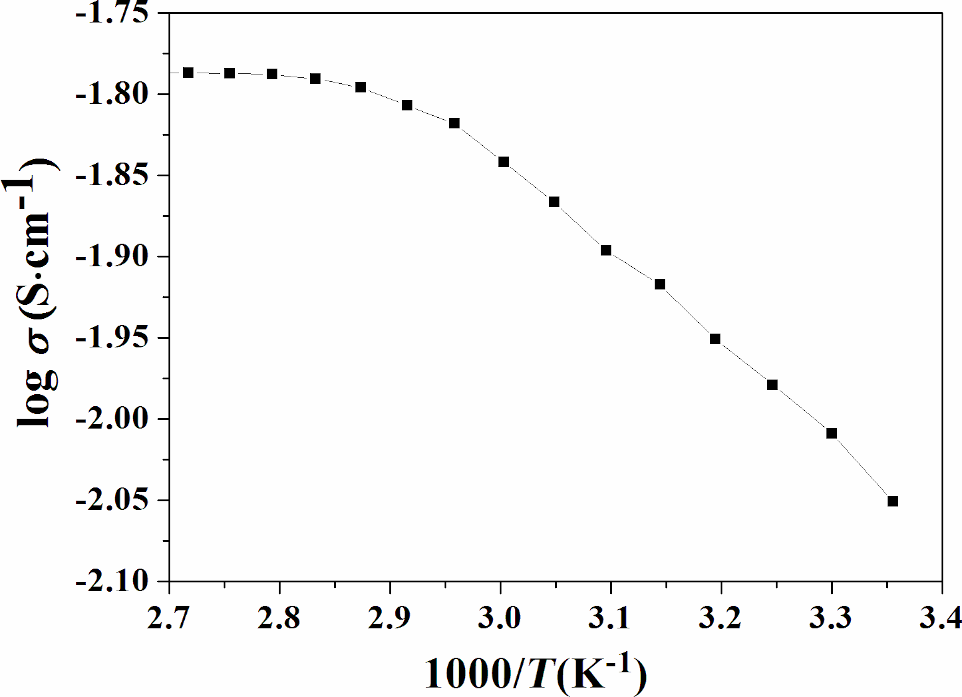
Variation of the electrical conductivity of an optimized polymer gel as a function of the temperature.

Figure 5 shows the temperature dependence plot of ionic liquid-based polymer gel electrolytes (log σ as a function of 1000/*T*). From the plot, it can be seen that the thermal dependence of the conductivity follows the Vogel–Tammann–Fulcher (VTF) equation, which is commonly used to explain the ion transport in amorphous polymer electrolytes [36–37]:


[2] σ=AT1/2exp(−BT−T0),

where *A* is a constant that shows the conductivity at an infinitely high temperature, the parameter *B* is the pseudo-activation energy and it is related to the critical free volume for ion transport, and *T*_0_ is a reference temperature, also called equilibrium glass transition temperature, which has a value close to the *T*_g_ values. In Figure 5, it can be clearly seen that the ionic conductivity increases with temperature, which can be understood in terms of the free volume model [10,38].

According to the free volume model, with a temperature increase the polymer can easily expand and produce free volume, which allows for the overall mobility of free ions and segmental motion of polymeric chains. As a result, there is an increase in the conductivity of the polymer gel electrolyte system. The VTF fitting parameters (i.e., *A*, *B*, and *T*_0_) were determined by using non-linear least square fitting of the data and are tabulated in Table 2.

**Table 2:** Non-linear fitting parameters from the VTF equation.

Sample	Parameters		

	*A* (S·cm^−1^)·K^−1/2^	*B* (eV)	*T*_0_ (K)

optimized polymer gel electrolyte	0.0368	0.27	272

**Figure 6:**
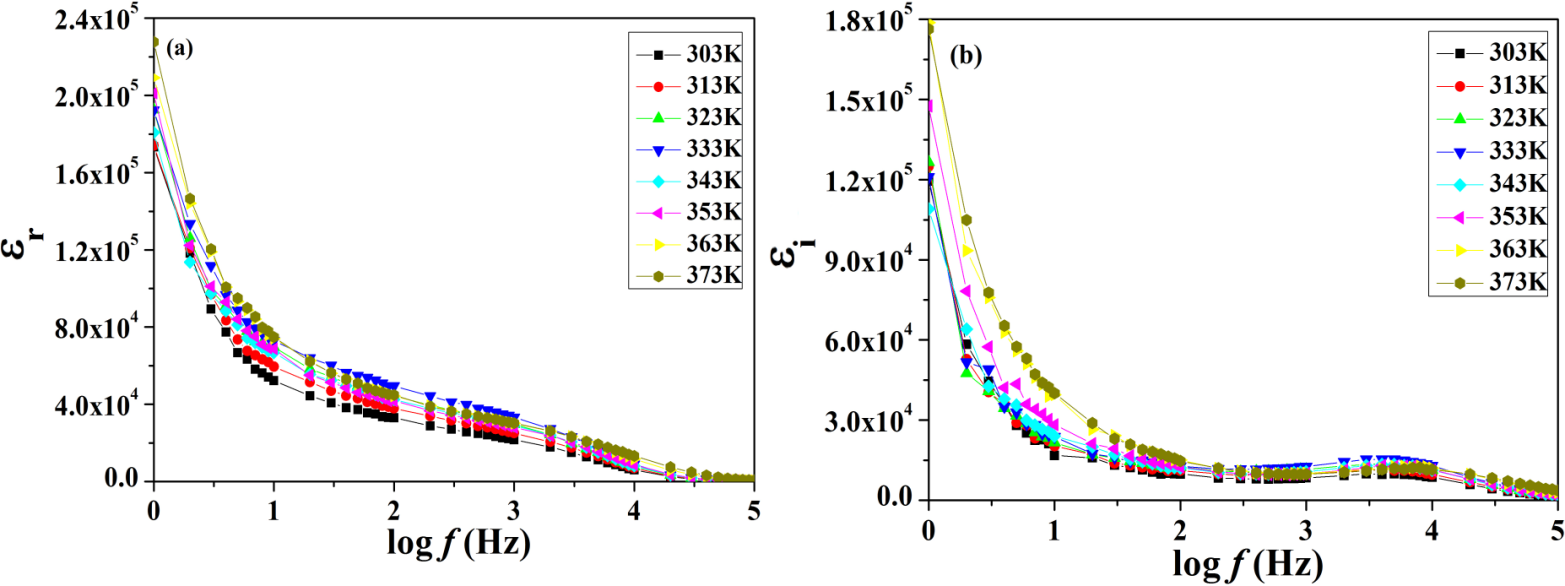
(a) Variation of the dielectric constant of a polymer gel electrolyte system as a function of frequency at various temperatures. (b) Variation of the dielectric loss of a polymer gel electrolyte system as a function of frequency at various temperatures.

Dielectric studies are some of the most important techniques to understand the effect of plasticizers, blending of polymers, inter-/intramolecular interactions, their transport mechanism, and relaxation behavior at a molecular level. Figure 6a and Figure 6b show the dielectric constant (ε_r_) and the dielectric loss (ε_i_) as a function of frequency at different temperatures for a polymer gel electrolytes system. It can be seen from Figure 6a that there is a large dielectric dispersion with increasing frequency values at a given temperature.

Dielectric dispersion which appears at higher temperatures is a measure of the dielectric relaxation which occurs due to the lagging time of rotation with respect to an external alternating field of side groups associated with the main chain. On the other hand, low-temperature dielectric dispersion is a measure of β-relaxation and is related to the micro-Brownian motion of the main chain due to the flexible nature of its constituent molecules. It is also observed from the figures that the decreasing pattern of the dielectric constant is more prominent toward lower frequencies as well as in the higher temperature domain. The decreasing pattern of the dielectric constant with increasing frequency is the most expected phenomenon of dielectric materials which mostly arises due to the dielectric relaxation that causes an anomalous dispersion. The orientational polarization, which depends on the molecular arrangement of dielectric materials, is the major cause of dielectric relaxation. At higher frequencies, the rate of rotational motion of polar molecules does not match the frequency of the applied field. Generally, it lags behind the frequency of the external field and leads to the decrease in the values of the dielectric constant with increasing frequencies [10]. From Figure 6b it can be seen that the larger values of the dielectric loss (ε_i_) are more prominent in lower frequencies as well as in higher temperature regions.

Mostly it arises due to the mobility of free charge carriers that exist within the dielectric materials. The general pattern observed in the plot of dielectric loss as a function of frequency suggests the existence of β-relaxation due to some local movements of side group dipoles. A further increase in the values of dielectric loss toward higher temperatures is also observed which indicates the presence of higher charge carriers due to the dissociation of salts and ion aggregates at higher temperatures.

**Figure 7:**
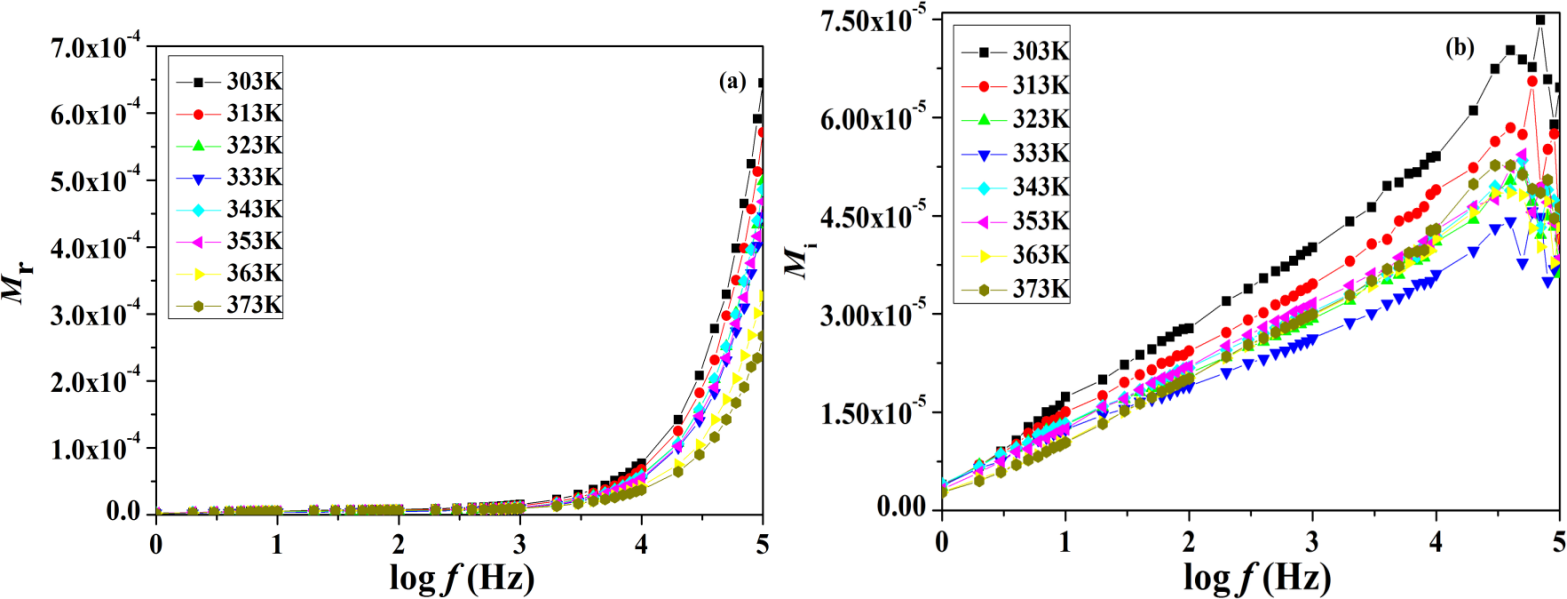
(a) Variation of the real part of the modulus of polymer gel electrolytes as a function of frequency at various temperatures. (b) Variation of the imaginary part of the modulus of polymer gel electrolytes as a function of frequency at various temperatures.

The analysis of the modulus is very useful in distinguishing between electrode polarization with that of other interfacial effects. Figure 7a and Figure 7b show the real part *M*_r_ and the imaginary part *M*_i_ of the electrical modulus at different temperatures for ionic liquid-based polymer gel electrolytes.

From both plots of the real and imaginary modulus, it can be seen that their values show an increasing pattern toward a higher frequency region for almost all temperatures, which may be due to the bulk effect. It is observed that the height of the peak decreases by increasing the temperature, which suggests a plurality of relaxation mechanisms. At low frequencies, the values of both real and imaginary moduli decrease to almost zero, indicating a negligible contribution of the electrode polarization phenomenon. The presence of a long tail in lower frequency regions also suggests that a larger capacitance is associated with the electrodes.

**Figure 8:**
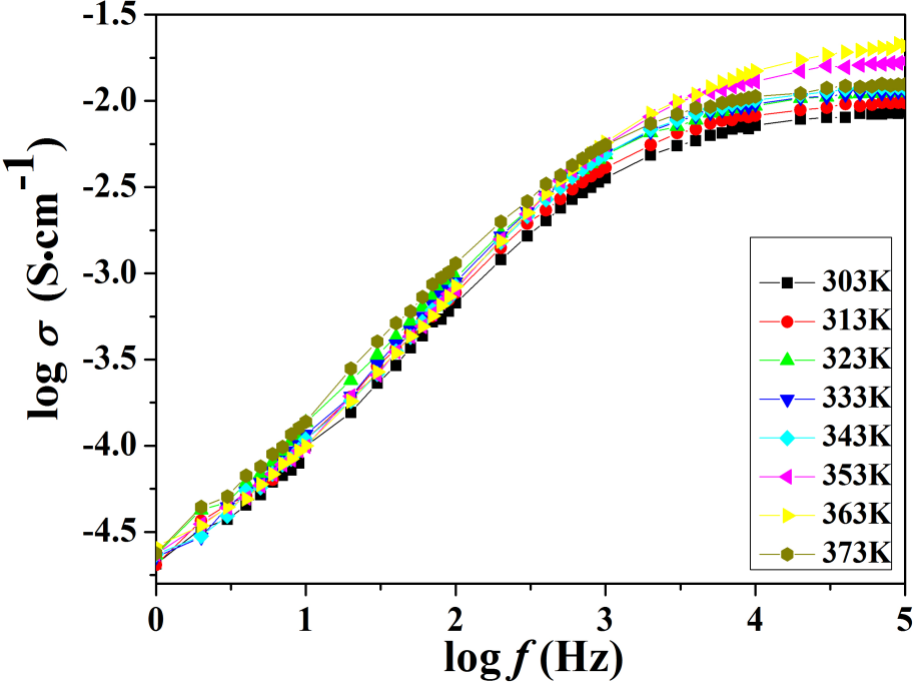
Variation of the ionic conductivity as a function of frequency at various temperatures.

Figure 8 shows the variation of the ac conductivity as a function of frequency at various temperatures for ionic liquid-based polymer gel electrolytes ({(PVdF-HFP)-[BDiMIM][Cl]} (4:6)) (20 wt %) + [PC-Mg(ClO_4_)_2_ (0.3 M)] (80 wt %). In general, the plot corresponds to the bulk relaxation phenomenon. The plot can be divided into two regions, namely low- and high-frequency regions. The low-frequency dispersion region describes the electrode–electrolytes interfacial phenomenon (or space charge polarization). As the frequency decreases, more and more charge accumulation occurs at the electrode and electrolyte interfaces, which leads to a decrease in the number of mobile ions and eventually to a drop in the conductivity at low-frequency values. In the high-frequency region, the mobility of charge carriers is high and hence the conductivity increases with frequency. The extrapolation of the plateau region to zero frequency gives the value of the dc ionic conductivity.

It is also observed that the ionic conductivity of polymer gel electrolytes increases as the temperature increases. This is due to the dissociation of ion aggregates at higher temperatures.

**Figure 9:**
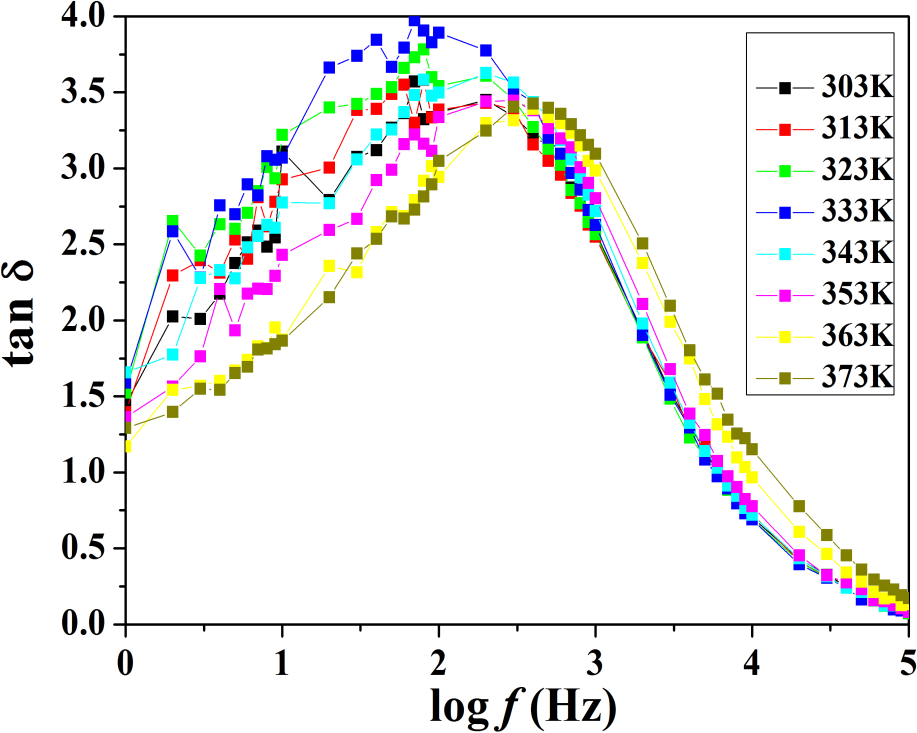
Variation of the loss tangent (tan δ) as a function of frequency at different temperatures.

Figure 9 shows the variation of tan δ as a function of frequency for an optimized composition of polymer gel electrolytes at different temperatures.

From Figure 9 it can be seen that the values of tan δ increase as the frequency increases, it reaches a maximum value, and finally decreases. Further, it can be seen that the maximum value of tan δ gets shifted toward the higher frequency side as the temperature increases, which is attributed to the enhancement of the number of charge carriers available for conduction. The results obtained from this study are in good agreement with the results of dielectric and conductivity methods.

**Figure 10:**
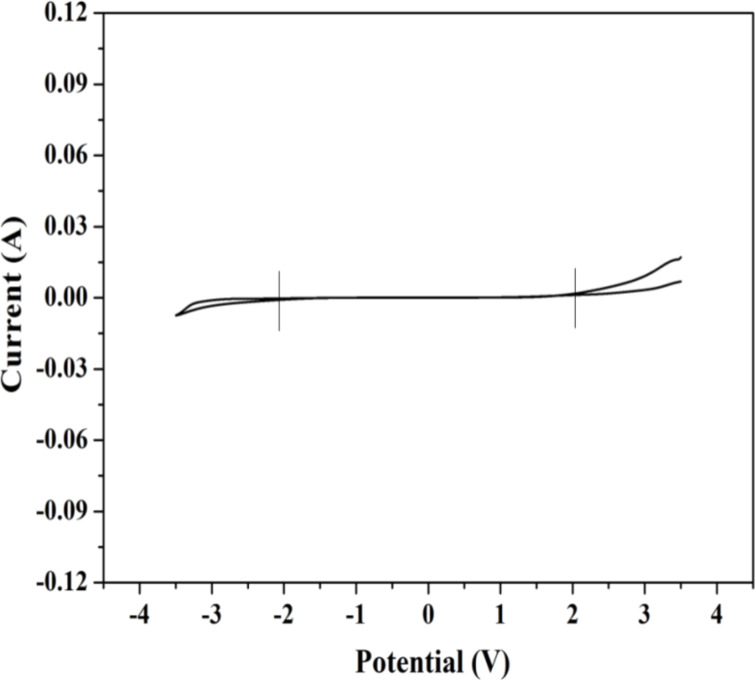
Linear sweep cyclic voltammetry curve of polymer gel electrolytes (cell stainless steel | polymer gel electrolyte | stainless steel) recorded at room temperature at a scan rate of 5 mV·s^−1^.

The electrochemical potential window measurement is one of the most important techniques that confirms the potential stability range of polymer gel electrolyte materials. From this study, the working voltage of synthesized electrolytes can also be determined by this method. In the present work, the electrochemical stability of polymer gel electrolytes has been recorded by using cyclic voltammetry. Figure 10 shows the *I*–*V* characteristics of the optimized polymer gel electrolytes which was carried out at a scan rate of 5 mV·s^−1^.

The plot clearly reflects that there is a gradual increase in the values of current with an increase in voltage up to certain values of potential. Afterward, there is an abrupt increase in the current value recorded which represents a working voltage, or potential window, of the synthesized polymer gel electrolyte. In the present study, the potential window was found to be in the order of ≈4.0 V.

**Figure 11:**
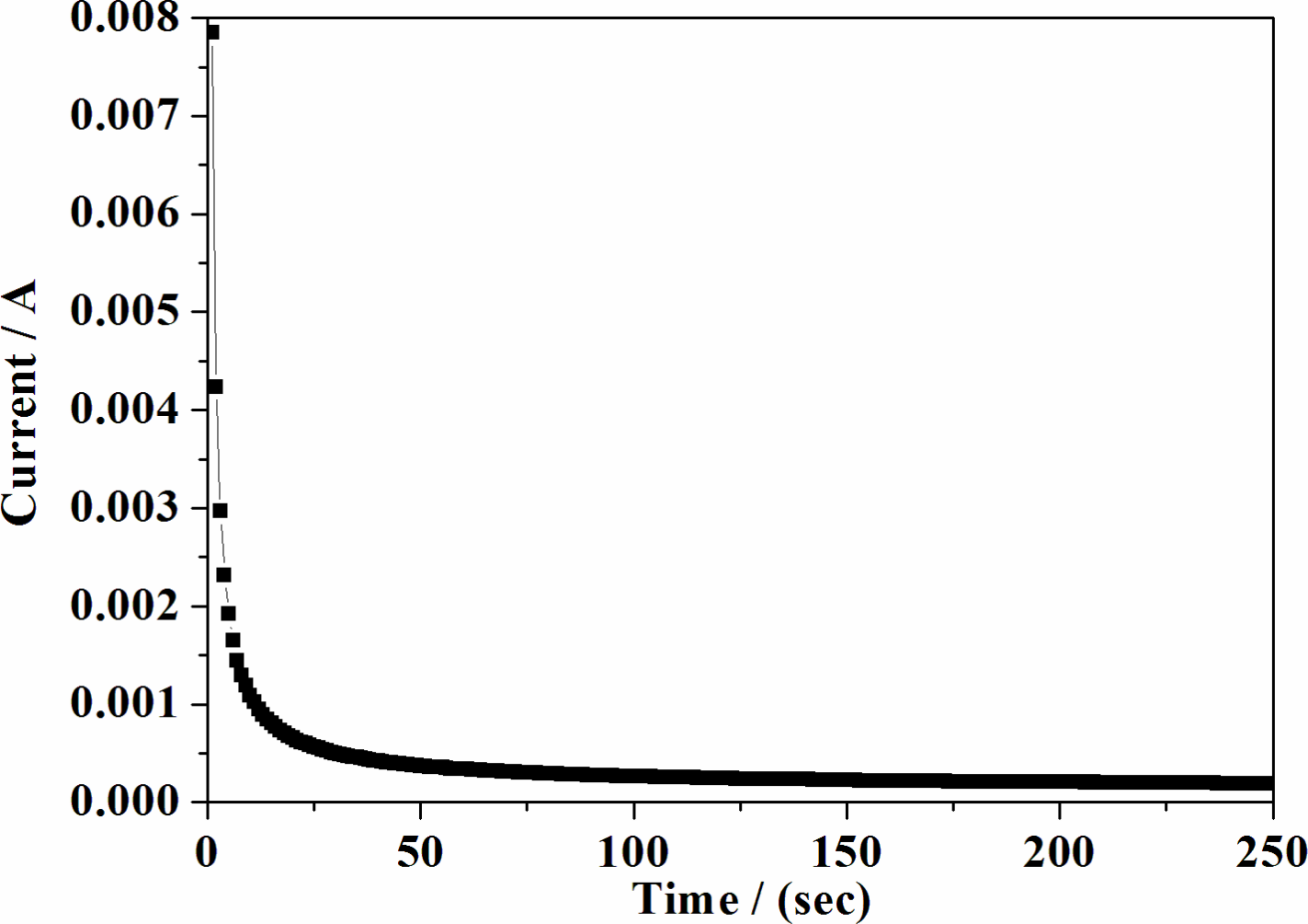
A dc polarization curve as a function of time for a polymer gel electrolyte.

The total ionic transference number of the prepared electrolyte system was calculated by using the dc polarization technique described in the Experimental section. The dc polarization curve of an ionic liquid-based polymer gel electrolyte system is shown in Figure 11. According to this method, the optimized film of a polymer gel electrolyte is sandwiched in between two symmetrical stainless steel (SS) electrodes and the film is polarized by applying a potential of 1.0 V. Thereafter, the resultant current as a function of time is monitored. In order to calculate the values of the ionic transport number, Equation 2 was used and this value was found to be 0.91. This means that the total conductivity in the present electrolyte films is primarily due to ions, and the electron contribution to the total conductivity is negligible.

## Conclusion

The present study was mainly focused on the development of polymer electrolytes with acceptable ionic conductivity values which can be suitable for energy storage applications. For this purpose, the ionic liquid [BDiMIM][Cl] was introduced in the polymer system. Along with the ionic liquid, magnesium salt was also used to prepare the final polymer films. The prepared films possess good dimensional and mechanical stability with acceptable parameters. Impedance spectroscopic studies confirmed that there is an increase in the room-temperature ionic conductivity of the prepared electrolyte films when the ionic liquid content is increased. The highest room-temperature ionic conductivity was found to be in the order of ≈8.9 × 10^−3^ S·cm^−1^ for the optimized system. The XRD data were also in line with the impedance data, confirming the increase in the amorphization of the system. The SEM images reveal the porous texture of the films, which have good liquid retention capability and support the electrical conductivity through their polymeric matrices. The electrochemical stability window of the prepared films was found to be 4.0 V. The ionic transference number also shows that the contribution of the electronic conductivity is negligible with respect to the total conductivity, and the prepared films are ionic in nature. All the results support that the prepared electrolyte has full potential to be used in high-performance electrochemical applications.

## Experimental

### Materials

The ionic liquid [BDiMIM][Cl], host polymer PVdF-HFP with an average molecular weight of 400000 (MW), and salt magnesium per chlorate Mg(ClO_4_)_2_ were all obtained from Sigma Aldrich, Germany. The plasticizer propylene carbonate was purchased from Loba Chemie, and the solvent acetonitrile (ACN) was obtained from Merck. All the chemicals were used without further purification.

### Preparation of IL-polymer gel electrolyte membranes

The electrolyte films were synthesized by using the standard solution casting technique. Details can be seen in our previous articles [10–11]. Briefly, different concentrations of the salt Mg(ClO_4_)_2_ were dissolved in the plasticizer PC. Different weight percentages of PVdF-HFP and ionic liquid were separately dissolved in a common solvent (i.e., ACN). The optimized solution of the liquid electrolyte was then mixed with the polymer/ionic liquid solution in different weight ratios and magnetically stirred for ≈9 h. The final optimized polymer electrolyte composition was ({(PVdF-HFP)-[BDiMIM][Cl]} (4:6)) (30 wt %) + [PC-Mg(ClO_4_)_2_ (0.3M)] (70 wt %). The optimized viscous mixture was then casted over glass Petri dishes and films were air dried at room temperature. After two weeks, free-standing polymer gel electrolyte films of ≈400–500 µm thickness were obtained.

### Measurements and characterization

X-ray diffraction studies of the polymer electrolyte films were recorded using a Bruker D8 Advance diffractometer with the Bragg angle (2θ) ranging from 10 to 60° and under Cu Kα radiation. The scan rate was fixed to 5°·min^−1^. The surface morphology of the synthesized polymer electrolyte films was recorded by using a scanning electron microscope (JEOL JXA - 8100 EPMA).

The ionic conductivity of any polymer gel electrolyte film plays an important role, especially at the time of fabrication of the devices. In the present study, σ was recorded by using complex impedance spectroscopic techniques, carried out by an LCR Hi-Tester (HIOKI-3522-50, Japan) in the frequency range of 100 kHz to 1 Hz with a signal level of 10 mV. The ionic conductivity was calculated by using following equation:


[3] $$\sigma =\frac{1}{R_{\text{b}}}\times \frac{l}{A},$$

where *l* is the thickness of the sample, *A* is the geometric surface area, and *R*_b_ is the bulk electrolyte resistance which is measured by using Nyquist plots. For conductivity measurements, the samples were cut into uniform sizes and sandwiched between two stainless steel electrodes.

Electrochemical properties, such as the electrochemical potential window and the total ionic transference number (*t*_ion_) of the polymer gel electrolyte films were examined by using the electrochemical analyzer CHI 608C (CH Instruments, USA). The dc polarization technique was used to determine *t*_ion_ in which a voltage of 1.0 V was applied across the polymer gel electrolyte film sandwiched between two stainless steel electrodes. The total ionic transference number was calculated by using the following equation:


[4] $$t_{\text{ion}}=\frac{i_{\text{T}}-i_{\text{e}}}{i_{\text{T}}},$$

where, *i*_T_ is the initial electronic current and *i*_e_ is the residual electronic current [9].
